# Contribution of Erythrocyte Sedimentation Rate to Predict Disease Severity and Outcome in COVID-19 Patients

**DOI:** 10.1155/2022/6510952

**Published:** 2022-08-11

**Authors:** Celali Kurt, Arzu Altunçeki̇ç Yildirim

**Affiliations:** Department of Infectious Diseases and Clinical Microbiology, Faculty of Medicine, Ordu Üniversity, Ordu, Turkey

## Abstract

**Aim:**

The use of erythrocyte sedimentation rate (ESR) in coronavirus disease 2019 (COVID-19) to determine disease severity and prognosis is limited. This study aimed to interrogate the diagnostic and prognostic role of ESR compared to other acute-phase reactants.

**Method:**

This retrospective cross-sectional study included 493 confirmed and hospitalized adult COVID-19 patients. Pneumonia, radiological severity, oxygen, intensive care requirements, mortality, ESR, and other acute-phase reactant values were recorded. Logistic regression and ROC analysis identified the effect of ESR on mortality and the sensitivity and specificity of the optimal cutoff values of ESR for the prediction of pneumonia, intensive care needs, and mortality and compared these with values for CRP.

**Results:**

Of patients, 346 (70.2%) had pneumonia, 98 (19.9%) required intensive care, 183 (37.1%) required oxygen support, and 62 (12.6%) died. ESR data were obtained for 278 patients. Among patients, 80.2% had ESR above 20 mm/h, with a median value of 53 (interquartile range: 49). ESR was higher among those with pneumonia (*p* < 0.001), requiring oxygen (*p* < 0.001), and requiring intensive care (*p*=0.003) compared to those without these, and in exitus patients (*p*=0.043) compared to survivors. Logistic regression analysis identified that ESR did not impact mortality. ROC analysis found the AUC, cutoff, sensitivity, and specificity results of ESR for pneumonia were 0.827, 37 mm/h, 77%, and 78%; for intensive care were 0.625, 50 mm/h, 74%, and 52; and for mortality were 0.606, 51 mm/h, 71%, and 49%, respectively. However, ROC analysis values for CRP were superior to ESR for all these categories.

**Conclusion:**

ESR increased in COVID-19 patients in the presence of pneumonia and severe disease; however, it was not prognostic. Sensitivity and specificity values for pneumonia, intensive care requirements, and mortality were lower than those for CRP.

## 1. Introduction

Due to coronavirus disease 2019 (COVID-19), a process deeply affecting the whole world in terms of individual and public health and social and economic aspects has lasted nearly two years, and it was declared a pandemic by the World Health Organization. More than 250 million people have been infected, and more than 5 million have died [1]. Intense studies about this pandemic's prevention routes, diagnosis, prognosis, and treatment continue worldwide. Among laboratory parameters varying in COVID-19, the most notable are increases in inflammatory markers, disruption of coagulation parameters, increases in nonspecific tissue injury markers, and hematological changes. Disruption of laboratory parameters is more pronounced in severe disease situations [2]. Attempts were made to identify clinical and laboratory parameters for determining the prognosis of the disease, and many studies investigated the correlation of acute-phase reactants with disease severity and mortality [3, 4]. Although erythrocyte sedimentation rate (ESR), which has been used in practice for many years, has been included among laboratory data in many COVID-19 studies, its benefits and necessity have not been sufficiently researched. Our study examined the correlation of ESR with disease severity and mortality in COVID-19 and the superiority of C-reactive protein (CRP) to interrogate its necessity.

## 2. Materials and Methods

The study included patients over 18 years admitted to the hospital with COVID-19 diagnosis confirmed with polymerase chain reaction (PCR) or with COVID-19 diagnosis using PCR after admission for other reasons from 15 March 2020 to 31 April 2021. PCR test was performed with real-time reverse transcriptase (Roche LightCycler® 480, Mannheim, Germany). Patient age, sex, ward of admission (floor, intensive care), presence of pneumonia, oxygen requirements, pulmonary imaging, laboratory findings, and outcome (discharge or exitus) were retrospectively determined from the hospital information system and the national health data records system.

Pneumonia diagnosis was decided with pulmonary radiography and computed tomography (CT) for those with the radiological investigation and clinical and biochemical findings for those without imaging. Patients with pneumonia identified on tomography had radiological severity rated from 1 to 5. Ratings were 1 for the involvement of less than 10% of pulmonary parenchyma, 2 for 10–30% involvement, 3 for 30–50% involvement, 4 for 50–75% involvement, and 5 for more than 75% involvement. This rating was made by a single clinician who was not a radiologist (infectious disease expert assigned to diagnosing and treating COVID-19 patients). The scoring did not note the nature of involvement (like ground glass and consolidation). Radiological scoring was performed before examining the patients' biochemical and clinical features. Additionally, severe pneumonia was assessed as having more than 50% involvement (rating 4 and 5), while mild pneumonia was assessed as less than 50% involvement (rating 1, 2, and 3).

Sedimentation, CRP, procalcitonin, and ferritin maximum values and definite lymphocyte count minimum values were recorded from laboratory data.

The Statistical Package for Social Sciences (IBM SPSS for Mac 26.0) program version 26 and Jamovi software version 2.2.3 were used for the statistical analysis. The chi-square analysis was used to compare categorical variables, and the normality test for quantitative variables was performed with the Shapiro–Wilk test. A comparison of two independent groups with normal distribution was made by Student's *t*-test, while the comparison of two independent groups without normal distribution was made by the nonparametric Mann–Whitney *U* test. The comparison of more than two groups was made by the Kruskal–Wallis test, and the comparison of dependent groups was made by the nonparametric Wilcoxon test. The level of significance was defined as *p* < 0.05. Univariate logistic regression analysis was performed to identify independent variables affecting the dependent variable of mortality, and multivariate binary logistic regression analysis was performed to identify variables significant in this analysis. The Spearman correlation test was performed for correlation analysis of groups not abiding by the normal distribution. Cutoff values, sensitivity, and specificity values for CRP and sedimentation parameters were calculated with receiver operating characteristic curve (ROC) analysis.

The study was approved by Ordu University Clinical Research Ethics Committee and conducted according to the principles of the Helsinki Declaration.

## 3. Results

The study included 493 patients. The median age of patients was 58 years (range 18–100), with 247 women included in the study (50.1%). Of patients, 346 (70.2%) had pneumonia diagnosis. Ninety-eight patients (19.9%) required intensive care, and 183 (37.1%) required oxygen support. All patients with pneumonia diagnosis had tomography. Of the 147 patients without pneumonia, 107 had tomography taken. Among the 40 patients without CT, 10 had pulmonary radiographs and 30 had no pulmonary imaging.

Of the patients, 62 (12.6%) died. The distribution of exitus patients is shown in Table 1. In the group with severe pneumonia, mortality was 34.6% (27 exitus/78 patients), while it was identified as 9.3% (35 exitus/375 patients) for the group with mild-moderate pneumonia (*p* < 0.001). 5 patients died without pneumonia; 4 had advanced malignancy, while 1 was an immobile patient with an advanced neurological disease who died due to gastrointestinal hemorrhage.

The comparison of laboratory data for patients according to outcome is given in Table 2.

Among patients, only 278 had sedimentation data. These 278 patients had a median ESR of 55.5 ± 33.2, with a median value of 53 (interval 2–150, interquartile range (IQR): 49). When the normal upper limit is accepted as 20 mm/h, 223 patients (80.2%) had high ESR values. Among those with ESR data, 23 of 47 patients without pneumonia (48.9%) had ESR above 20 mm/h, with a mean ESR of 25.1 ± 21.1 and median value of 20 mm/h (interval 2–86, 95% confidence interval 19.1–31.2, IQR: 25). Of the 231 patients with pneumonia, 200 (86.6%) had ESR above normal.

There was a positive, significant, low correlation between ESR value and age (*p* < 0.001, Spearman correlation coefficient: 0.224). ESR values were significantly higher for those with pneumonia, severe pneumonia, oxygen requirements, and intensive care needs compared to patients without these and among exitus patients compared to surviving patients. The group with cancer and rheumatological diseases that may affect ESR did not have significant differences compared to the group without these factors. The comparison of ESR values in various groups is given in Table 3.

Of the 278 patients with ESR examined, 64 had more than one result. Of these, 35 had the ESR test twice and 29 had it three times. For all patients tested twice, the final test was lower than the first, while for all patients tested three times, the final ESR value was lower than the maximum value. The final ESR median value was 44 (IQR: 38), while the maximum ESR median value for the same patients was 77 (IQR: 41) (*p* < 0.001). Among the 64 patients with ESR examined more than once, 12 entered intensive care and only one died. There was a significant difference between the maximum ESR values for patients requiring and not requiring intensive care, with no difference identified between final ESR values (50.5 IQR: 35.5 and 29.5 IQR: 28.8, *p*=0.14).

Variables (intensive care need, pneumonia, more than 50% involvement in CT, age over 60 years, CRP, lymphocyte count, sedimentation, and ferritin) which were significant in univariate logistic regression analysis to identify factors affecting the outcome (exitus and survival) underwent multivariate logistic regression analysis. Sex and oxygen requirements were not significant in univariate analysis and not included in the model for multivariate analysis. Intensive care needs, low lymphocyte count, and high ferritin had significant correlations with mortality; however, age, pneumonia, CT severity, CRP, and sedimentation were not significant (Table 4).

As a result of the ROC analysis, it was seen that the predictive value of ESR was the highest in the presence of pneumonia. However, lower AUC values than CRP were found in this subject and other subjects, such as the need for intensive care and death. Cutoff, AUC, sensitivity, and specificity values of ESR and CRP are shown infigures 1–3 and Table 5.

## 4. Discussion

In our study, ESR was significantly higher in patients with COVID-19 having pneumonia, oxygen requirements, and intensive care needs than in those without. There was no significant difference identified between the sexes. The mean ESR values of exitus patients and those with widespread pneumonia involvement (>50%) on CT were higher than survivors and those with milder involvement, respectively. Among the other parameters for exitus and surviving patients, mean CRP, procalcitonin, ferritin, and lymphocyte counts were higher than ESR with a significant degree of difference (for ESR P: 0.043, others *p* < 0.001). Multivariate binary logistic regression analysis found that lymphocyte count, ferritin level, and intensive care needs were significant parameters predicting death, while CRP, ESR, age over 60 years, and severe involvement in CT were not significant.

ROC analysis showed that the most beneficial point of ESR for contributing to diagnosis was its predictive power for the presence of pneumonia (AUC: 0.827, sensitivity and specificity values of 77% and 78% for 37 mm/h cutoff value). However, even for this topic, CRP was identified to be more beneficial. In terms of mortality and intensive care requirements, ESR achieved a low AUC value, and CRP predicted higher sensitivity and specificity in these situations.

Despite several studies assessing acute-phase responses in COVID-19 disease since the start of the pandemic, including ESR data, the number of studies investigating ESR in depth is very few. Our study investigated ESR values in different stages and radiological severity levels of the disease and revealed new findings in comparing diagnostic benefits with other acute-phase reactants. In daily practice, parameters like CRP, which is more dynamic with a shorter half-life, ferritin, fibrinogen, procalcitonin, and d-dimer, are more commonly used [5, 6]. However, high ESR rates were reported in the first case series. A 28 case series including 24 pneumonia cases from January and February 2020 at the start of the pandemic found that the mean ESR was 51.79 ± 32.02; when <15 mm/h is accepted as normal, 25 patients had increased levels (89.3%) [7]. Again, a meta-analysis from the early period of the pandemic showed laboratory parameters were more clearly disrupted in severe and mortal diseases, and ESR was increased [2]. Studies are reporting that the increased sedimentation is more pronounced in severe diseases. A study assessing data from 148 confirmed COVID-19 patients, performed in Turkey by Kaya et al., found that median ESR was significantly higher in patients with severe/critical disease (66.5 compared to 35.5, *p* < 0.001). In this study, ESR was identified to be an independent factor in predicting severe disease and death, with the cutoff value of 52.5 mm/h having sensitivity and specificity of 65.5% and 76.3% for severe disease and the cutoff value of 56.5 mm/h having sensitivity and specificity of 66.7% and 72.5% for mortality [8].

In China, a meta-analysis of 28 studies by Zhang et al., including 4663 cases, identified ESR increases in 61.2% of cases (95% CI 41.3–81.0%). In this study, CRP, LDH elevation, and lymphopenia were compared in groups with and without severe disease and found to comprise risk for severe disease. However, sedimentation was not investigated between the groups [9]. Another meta-analysis by Pormohammad et al., including 80 studies with 61,742 confirmed COVID-19 cases found that only 320 cases from 4 studies had sedimentation results recorded with the mean value of 44 mm/h (95% CI 46–57) [10]. A review by Lapic et al. including a total of 819 patients, including 358 severe cases, reported that despite all heterogeneity and limitations of the studies, apparent elevations in ESR were associated with severe COVID-19 patients compared to nonsevere cases. Despite low analytical and diagnostic specificity, they interpreted that it may benefit disease follow-up [11].

A simple, cheap, and rapid laboratory test has been commonly used for nearly one hundred years to identify the sedimentation rate of erythrocytes, and the discovery of ESR dates back to 1897 [12]. It is based on the increase in the sedimentation rate of erythrocytes linked to increased plasma proteins in inflammatory, infectious, and malignant diseases. However, this test is known with low specificity increasing with female sex, age, pregnancy, anemia, obesity, and chronic diseases [13]. As a result, formulas including age and sex are recommended and used to calculate normal ESR [14]. In practice, generally, CRP and ESR tests are requested together. CRP may follow a more rapid fall with the reduction in inflammation due to its shorter half-life and is beneficial for rapid assessment of treatment response. CRP and ESR are mostly correlated, but situations with incompatibility are known [15]. A meta-analysis indicated that using both parameters to assess different acute inflammatory situations is common and beneficial. Despite some incompatible results, diagnostic contributions are similar, while using them together was identified to increase diagnostic accuracy [16]. A study in Spain examined ESR and CRP values in participants in a volunteer group of the general adult population without known inflammatory disease; 74.9% were found to have normal values, and the incompatibility between these two values was examined. While the incompatible model with high ESR and normal CRP was associated with older age, the model with high CRP and normal ESR was associated with higher body mass index [17].

Apart from hematological and biochemical parameters, radiological methods are used to determine the disease severity of COVID-19. As the severity of the disease increased, along with the disruption of laboratory values, there was an increase in radiological involvement scores. CT is accepted as the most effective method to identify pulmonary abnormalities. Various radiological weighted scoring systems are used for CT assessment, and these were found to have sensitivity from 80 to 83 and specificity from 82 to 100 for the detection of disease severity [18]. Another study of 83 COVID-19 pneumonia patients, including 25 serious/critical cases and 58 nonsevere cases, identified a significant difference in the laboratory values of severe patients, especially in CT scores [19]. Typical COVID-19 pneumonia is observed on CT as bilateral frosted glass opacity, consolidations, and air bronchograms in the subpleural areas and lung basal areas [20]. A study assessing 165 patients in which 30 were severe found that crazy-paving patterns, linear opacities, air bronchograms, and the presence of extrapulmonary findings were correlated with severe/critical disease. A radiological scoring system with a total of 96 points was used, and severe patients had a mean score of 63.9 while mild patients had a mean score of 35.6 (*p* > 0.01), and a score of 38 was identified to predict severe disease [21]. In our study, in the group with radiological weighting in the 5th category (the group with 75% or more involvement), 8 out of 14 patients died (57.1%). In groups with mild pneumonia (groups 1, 2, and 3) and with severe pneumonia (groups 4 and 5), mortality and ESR rates were 9.3% and 52.3 mm/h and 34.6% and 71.4 mm/h, respectively (*p* < 0.001).

This study has several significant limitations. Being a retrospective study prevented the study's prospective design and planning for laboratory parameters in this way.

Additionally, different pandemic clinics in our hospital were managed by different branches and specializations (chest diseases, internal medicine, infectious diseases, and intensive care specialists), which caused differences in the use of laboratory tests. For this reason, it appears that the differences in individual approaches of some specialist branches and clinicians caused data loss, especially about ESR. ESR tests were from the periods when patients were monitored in the ward and were admitted to primary intensive care. The tertiary intensive care clinical follow-up protocol does not include ESR, so data come from the period when patients were monitored on the ward. This situation prevented serial ESR monitoring of many patients and detecting values from the most severe period of the patient's clinical progression.

Additionally, the frequency of requesting acute-phase reactants was variable according to the clinical severity of the patient and the doctor. These prevented the chronology for ESR and other acute-phase reactants from being simultaneous. The most pathological values obtained during the duration of follow-up for the patients were assessed. ESR data were obtained in the first 15-day monitoring duration according to most patients' admission and hospitalization duration. The days when disease symptoms were noted and microbiological diagnoses were received are unclear. As ESR is a test with low specificity affected by many physiological and pathological situations, it is necessary to be careful when interpreting data. When anemia, comorbid inflammatory, and other chronic diseases that may affect ESR are commonly seen at advanced ages, COVID-19 is more severe and deadly, which may overestimate the ESR and COVID-19 relationship. Despite all these limitations, it is considered that the degree of benefit of ESR compared to other acute-phase reactants, especially CRP, was revealed to determine the severity and progression of the disease. However, there is a need to identify laboratory parameters with more specific and prognostic features that can be used to determine the severity of COVID-19.

In conclusion, increases in acute-phase reactants, disruptions in hematological and coagulation parameters, and nonspecific tissue injury markers are observed in COVID-19. The disruptions in laboratory parameters are more pronounced in severe patients. Along with radiological methods, mostly CRP, ferritin, procalcitonin, d-dimer, fibrinogen, interleukin-6 levels, leukocyte, lymphocyte, and platelet counts are used for the detection of disease severity and follow-up. Many publications report that ESR increases in COVID-19 and the changes are more pronounced with other laboratory values in severe diseases. However, the extra contribution in addition to other parameters was not questioned. In our study, although ESR was shown to be significantly increased in groups with COVID-19 pneumonia, severe pneumonia, and intensive care requirements and who died, it was not prognostic and had less benefit for diagnosis and follow-up than CRP. It will be beneficial to follow up along with other parameters in selected nongeriatric COVID-19 patients without comorbid disease; however, it was concluded that it does not provide additional contributions in routine use.

## Figures and Tables

**Figure 1:**
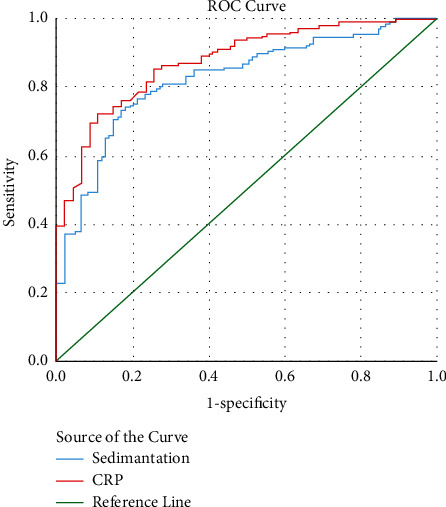
ROC curves for the prediction of pneumonia by sedimentation and CRP.

**Figure 2:**
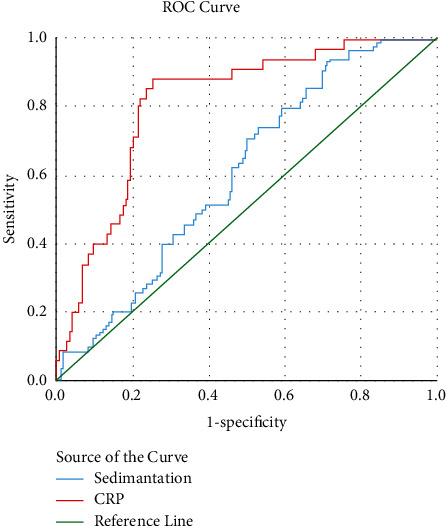
ROC curves for the prediction of mortality by sedimentation and CRP.

**Figure 3:**
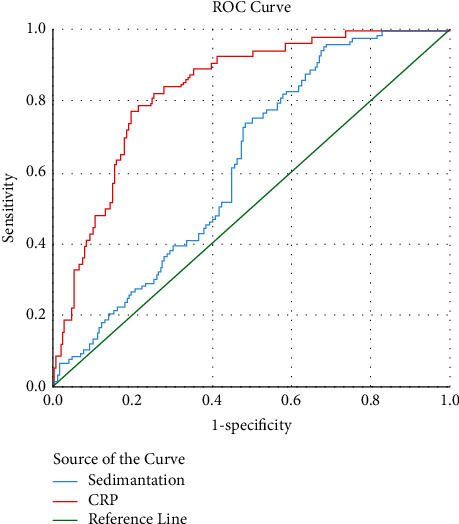
ROC curves for the prediction of intensive care needs by sedimentation and CRP.

**Table 1:** Distribution of exitus patients.

		N	Exitus N (%)	*p value*
Pneumonia	Yes	346	57 (16.5)	<0.001
	No	147	5 (3.4)	
Oxygen requirements	Yes	183	62 (33.9)	<0.001
	No	310	0	
Intensive care need	Yes	98	58 (59.2)	<0.001
	No	395	4 (1)	
Age^*∗*^	18–40	124	0 a	<0.001
	41–50	63	2 (3.2) a,b	
	51–60	82	5 (6.1) a,b	
	61–70	79	9 (11.4) b	
	>70	145	46 (31.7) c	
CT severity^*∗*^	0	107	5 (4.7) a	<0.001
	1	62	2 (3.2) a,b	
	2	70	5 (7.1) a,b	
	3	136	23 (16.9) b,c	
	4	64	19 (29.7) c,d	
	5	14	8 (57.1) d	
Sex	Women	247	31 (12.6)	0.986
	Men	246	31 (12.6)	

^*∗*^Groups without statistical differences at *p* : 0.05 significance level shown with the same letter. CT: computed tomography.

**Table 2:** Comparison of laboratory data according to the outcome.

	Surviving N median (IQR)	Exitus N median (IQR)	*p value*
CRP (mg/dL)	427 4.31 (11.43)	62 17.95 (12.03)	<0.001
Lymphocyte (/*μ*L)	428 920 (832)	61 280 (260)	<0.001
Ferritin (*μ*g/L)	372 308.5 (595.5)	58 1107 (1324.5)	<0.001
Procalcitonin (*μ*g/L)	326 0.08 (0.13)	60 1.65 (6.05)	<0.001
Sedimentation (mm/h)	243 51 (54.5)	35 66 (39)	0.043

SD: standard deviation; CRP: C-reactive protein; IQR: interquartile range.

**Table 3:** Comparison of sedimentation values in various groups.

		N	Sedimentation^†^ (median)	IQR	*p value*
Sex	Women	126	57.5	46.5	0.671
	Men	152	52	53.8	

Age groups^*∗*^	18–40	38	15.5 a	38.5	<0.001
	41–50	36	52 b	60.5	
	51–60	48	57.5 b	55	
	61–70	66	71.5 b	42	
	>70	90	52 b	44.3	

Age	<60	122	44.5	61.5	<0.001
	>60	156	64.5	47.3	

Pneumonia	Yes	231	62	47	<0.001
	No	47	20	25	

Oxygen requirements	Yes	123	67	37	<0.001
	No	155	38	58.5	

Intensive care needs	Yes	58	62	36.3	0.003
	No	220	48	57.5	

Outcome	Survived	243	51	54.5	0.043
	Exitus	35	66	39	

Factor affecting sedimentation	Yes	21	58	56	0.122
	No	257	52	50	

Pneumonia	Mild-moderate	221	48	56	<0.001
	Severe	50	75.5	27	

CT category^*∗*^	1	41	18 a	29	<0.001
	2	47	59 b	38	
	3	93	72 b	46	
	4	43	76 b	27.5	
	5	7	67 a,b	47	

^*∗*^Groups without statistical differences at *p* : 0.05 significance level shown with the same letter. CT: computed tomography; IQR: interquartile range; ^†^: mm/h.

**Table 4:** Analysis of factors affecting mortality.

	B	SE	Wald	Df	*p* value	Odds ratio	95% confidence interval	
							Lower	Upper
Intensive care needs	4.540	1.155	15.447	1	<0.001	93.652	9.736	900.891
Presence of pneumonia	1.280	1.513	0.716	1	0.397	3.598	0.185	69.835
Sedimentation	−0.019	.013	2.040	1	0.153	0.981	0.956	1.007
CRP	−0.020	0.040	0.241	1	0.623	0.980	0.906	1.061
Lymphocyte	−0.006	0.002	10.859	1	0.001	0.994	0.990	0.998
Ferritin	0.001	0.0002	3.920	1	0.048	1.001	1.000	1.001
More than 50% involvement in CT	−0.350	0.711	0.242	1	0.623	0.705	0.175	2.839
Age >60	0.750	0.901	0.692	1	0.406	2.117	0.362	12.388
Constant	−2.597	2.242	1.342	1	0.247	0.074		

Omnibus test of model coefficients: chi-square: 134.242, *p* < 0.001. −2 log-likelihood: 61.407; Cox & Snell *R* square: 0.413; Nagelkerke *R* square: 0.765; Hosmer and Lemeshow test sig: 0.999.

**Table 5:** Sensitivity and specificity values for CRP and sedimentation.

		AUC	SE	*P* value	95% confidence interval		Cutoff	Sensitivity %	Specificity %
					Lower	Upper			
Sedimentation^*∗*^	Pneumonia	0.827	0.030	<0.001	0.768	0.887	37	77	78
	ICU	0.625	0.036	0.003	0.555	0.696	50	74	52
	Exitus	0.606	0.044	0.043	0.519	0.692	51	71	49
CRP^†^	Pneumonia	0.89	0.015	>0.001	0.859	0.920	2.95	80	84
	ICU	0.872	0.018	>0.001	0.836	0.908	11.2	85	79
	Exitus	0.848	0.023	>0.001	0.804	0.893	14.5	80	80

AUC: area under the curve; SE: standard error; ICU: intensive care unit; ^*∗*^: mm/h; ^†^: mg/dL.

## Data Availability

The data used to support the findings of this study have not been made available because of provincial health directorate rules.
